# Spatiotemporal Variations in Microbial Community Structure and Assembly Mechanisms Within Recirculating Aquaculture Systems for Mandarin Fish (*Siniperca chuatsi*)

**DOI:** 10.3390/microorganisms14040794

**Published:** 2026-03-31

**Authors:** Zhengxi Wang, Decheng Pu, Peiyuan Li, Jishu Zheng, Dongsheng Li, Lin Zhou, Xiuli Wei, Lixiang Wang

**Affiliations:** Key Laboratory of Smart Agricultural Technology in the Southwest Mountains, Ministry of Agriculture and Rural Affairs (Co-Construction by Ministry and Province), Chongqing Academy of Agricultural Sciences, Chongqing 400715, China; pudc801@sina.com (D.P.); lpy0616@126.com (P.L.); imzhengjishu@163.com (J.Z.); dawson.lee@foxmail.com (D.L.); 1219228196@qq.com (L.Z.); wei77703863@126.com (X.W.); 13036328893@163.com (L.W.)

**Keywords:** *Siniperca chuatsi*, recirculating aquaculture system, microorganism, assembly mechanisms, co-occurrence network, spatiotemporal variations

## Abstract

Mandarin fish (*Siniperca chuatsi*) is a carnivorous fish species endemic to China with significant economic value. The Recirculating Aquaculture System (RAS) has exhibited promising application prospects in the culture of this species. However, the role of the succession patterns of microbial community structure in maintaining the ecological function and stability of this system remains poorly understood. Therefore, this study employed 16S rRNA high-throughput sequencing to analyze community characteristics, assembly mechanisms, co-occurrence networks, and potential functions across different functional zones and culture cycles. The results indicated that, temporally, alpha diversity decreased significantly during the T4 stage due to stress from nutrient accumulation and metabolic waste, accompanied by a distinct succession of dominant taxa. As the system entered the T5 stage, self-purification capacity improved, and microbial diversity gradually recovered. Spatially, significant differences in microbial composition were observed across environments, reflecting the strong influence of environmental specificity on community structure. Analysis of assembly mechanisms revealed that stochastic processes played a dominant role in driving the microbial community, particularly during the T3–T4 stages and within the YCS and TSC zones. Conversely, microbial dispersal was limited in the GC and LHC zones due to habitat barriers. Co-occurrence network analysis demonstrated that microbial interactions were predominantly competitive, with the network structure shifting from loose to modular over time. Spatially, differentiation arises due to varying functional requirements. Functional prediction identified chemoheterotrophy as the core metabolic function. Furthermore, the nitrogen transformation pathway shifted from predominantly denitrification to urea hydrolysis and nitrate reduction as the culture period progressed. These findings highlight the risk of nitrite and ammonia nitrogen accumulation in later stages and provide a theoretical basis for the optimization and management of RAS for Mandarin fish.

## 1. Introduction

Aquaculture has emerged as one of the fastest-growing animal food production sectors worldwide, playing a pivotal role in safeguarding global food security, alleviating pressure on wild fishery resources, and improving economic returns [1]. However, with the dramatic expansion of the industry scale, traditional aquaculture practices have exacerbated the competition for and consumption of natural resources, including land and freshwater [2]. Furthermore, large quantities of uneaten feed, fish excreta, and therapeutic chemical agents are directly discharged without sufficient treatment. This can easily lead to eutrophication of nearby natural water bodies, water quality deterioration, and damage to the benthic ecosystem [3]. In contrast, Recirculating Aquaculture Systems (RAS) offer an eco-friendly, resource-efficient, and intensive farming model [4]. By utilizing closed-loop circulation, RAS minimizes tailwater discharge. This effectively reduces water pollution while optimizing resource utilization [5]. At present, RAS has been widely commercially applied across the globe, especially in countries and regions with constrained land and water resources. Previous studies have documented that RAS has been extensively adopted for the hatching and grow-out production of *Salmo salar* in Northern Europe and North America [6]. In parts of Europe, this technology has also been widely applied in the intensive farming of species such as *Anguilla anguilla* [7]. In recent years, with the rapid development of facility fishery in China, the application of RAS in the culture of high-economic-value fish species including *Siniperca chuatsi* and *Micropterus salmoides* has also increased significantly [8].

The core concept of RAS involves processing water through physical, biological, and chemical treatments within a closed loop. This approach achieves water recycling while minimizing tailwater discharge [9,10]. Within this environment, the diversity and structure of microbial communities significantly influence water quality and fish health, including functional bacteria, probiotics, and pathogens [11]. Specifically, microorganisms associated with nitrification and denitrification, such as *Nitrosomonas*, *Nitrobacter*, and *Nitrospira*, remove excess nitrogen and phosphorus [12]. This biological process prevents water quality deterioration that would otherwise harm fish [13]. Furthermore, beneficial bacteria like *Bacillus* [14] and *Pseudomonas* [15] improve water quality by decomposing residual feed and feces, while simultaneously inhibiting the proliferation of harmful bacteria. Beneficial microorganisms colonizing the intestinal tract of fish, such as the genera *Butyricicoccus* [16], *Bifidobacterium* [17], and *Peptostreptococcus* [18], can significantly improve the growth performance and immunity of the host fish. Conversely, pathogens such as *Vibrio*, *Aeromonas*, and *Mycoplasma* can cause disease outbreaks [19]. Consequently, microbial communities are critical for maintaining the system’s ecological stability. However, current research on microbial communities within RAS remains subject to notable limitations. In terms of research subjects, most previous studies have focused on a single sampling site or specific host habitat, such as aquaculture water, biofilters, and the fish intestinal tract [20]. Yet independent analyses of these individual compartments have overlooked the microbial interactions and spatial continuity across distinct habitats. In terms of research methodologies, with the rapid development of sequencing technology, high-throughput sequencing based on 16S rRNA gene amplicon sequencing has increasingly become the mainstream approach for characterizing microbial community structures [21]. The primary advantage of this technology lies in its ability to accurately resolve the taxonomic composition and relative abundance of microbial communities, and it has been widely implemented in intestinal microbiota research. Nonetheless, there remains a paucity of comprehensive studies that treat all compartments of the RAS as an integrated organic whole. This makes it difficult to systematically evaluate the dynamic succession of microbial communities during system operation and their subsequent impacts on aquaculture production performance.

The Mandarin fish (*Siniperca chuatsi*) is an economically important freshwater species in China. Recently, the resolution of feed domestication challenges has driven rapid growth in the pellet-fed Mandarin fish aquaculture industry [22]. However, the domestication and culture of Mandarin fish are primarily conducted in traditional ponds. This method is inefficient, difficult to monitor, and prone to disease outbreaks and water pollution, which compromise fish quality [23]. As a closed, efficient, and sustainable intensive farming model, RAS effectively addresses these challenges, offering significant potential for pellet-fed Mandarin fish culture. Current research mainly focuses on dietary domestication [24], nutritional requirements [25], disease control [26], and breeding [27]. Although some studies have examined gut microbiota in RAS [28], systematic research on the characteristics, succession dynamics, and interactions of microbial communities within treatment systems and intestines under production conditions remains limited. Therefore, this study investigates four components of a Mandarin fish RAS: rearing tanks, biofilters, lift tanks, and intestines. Using 16S rRNA high-throughput sequencing, we analyzed microbial community composition, diversity, assembly mechanisms, co-occurrence networks, and function prediction across spatiotemporal scales. These findings reveal interaction patterns and succession dynamics, providing a scientific basis for optimizing Mandarin fish RAS management.

## 2. Materials and Methods

### 2.1. Construction of Recirculating Aquaculture Systems and Sampling Point Layout

**Figure 1:**
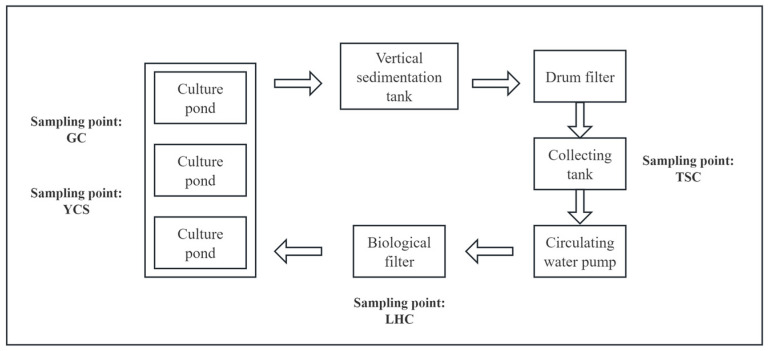
Schematic Diagram of the RAS for Mandarin Fish and Sampling Point Distribution.

The experiment was conducted at the Industrialized Agriculture R&D Center in Jiulongpo District, Chongqing. The Recirculating Aquaculture System (RAS) comprised three rearing tanks (diameter: 4 m; height: 1.5 m), a micro-screen drum filter, and a biofilter. Water circulated from the rearing tanks to a lift tank housing the micro-screen filter, passed through the biofilter, and returned to the rearing tanks to complete the cycle (Figure 1). The total water volume of the system was 75 m^3^. Feed-domesticated Mandarin fish were stocked at an average density of 25 kg/m^3^. Fish were fed Jieda extruded pellets (Guangdong, China) twice daily, with rations adjusted according to growth. During the experimental period, the dissolved oxygen in the recirculating aquaculture system was maintained above 8 mg/L, the water temperature was kept at 20–25 °C, and the pH level was maintained between 7.0 and 8.0. Regular water exchange was conducted, with a volume equivalent to 1% of the aquaculture tank capacity. Four specific components were designated as sampling sites: the Mandarin fish gut (GC), rearing tanks (YCS), lift tank (TSC), and biofilter (LHC). The study spanned from December 2024 to April 2025, during which the system maintained stable operation with continuous circulation. Water and gut samples were collected at the end of each month.

### 2.2. Sample Collection

Triplicate water samples were collected from the rearing tanks (YCS) (*n* = 3), lift tank (TSC) (*n* = 3), and biofilter (LHC) (*n* = 3). These samples were filtered sequentially through 0.45 μm and 0.22 μm membranes and then stored at −80 °C for DNA extraction. Additionally, Mandarin fish (Length: 10–18 cm, Weight: 16–140 g) were anesthetized with 100 mg/L MS-222. Surface sterilization was performed using 70% ethanol and sterile water. Intestines (GC) were aseptically dissected on ice. Intestinal contents from three fish were pooled into a single replicate. A total of 9 Mandarin fish were collected (*n* = 3) and stored at −80 °C for sequencing.

This experiment was sampled at 5 different time points. Each sampling session involved 12 samples, resulting in a total of 60 samples. Samples collected in December 2024 (T1), January 2025 (T2), February 2025 (T3), March 2025 (T4), and April 2025 (T5) were designated as GC1/YCS1/TSC1/LHC1, GC2/YCS2/TSC2/LHC2, GC3/YCS3/TSC3/LHC3, GC4/YCS4/TSC4/LHC4, and GC5/YCS5/TSC5/LHC5, respectively.

### 2.3. DNA Extraction and High-Throughput Sequencing

Microbial communities were analyzed using bacterial 16S rRNA gene amplicon sequencing. Total DNA from water samples was extracted using the E.Z.N.A^®^ Soil DNA Kit (Omega Bio-tek, Norcross, GA, USA). Total DNA from the intestinal tract of mandarin fish was extracted using the TIAN Microbe Magnetic Envir-DNA Kit4 (Tiangen, Beijing, China). Its concentration was measured using a Nano Drop 2000 spectrophotometer (Thermo Fisher, Waltham, MA, USA). Genomic DNA integrity was verified via 1% agarose gel electrophoresis to ensure quality for PCR amplification. The V3–V4 hypervariable zones of the bacterial 16S rRNA gene were amplified using specific barcode-tagged primers: 338F: 5′-ACTCCTACGGGAGGCAGCA-3′, 806R: 5′-GGACTACHVGGGTWTCTAAT-3′. PCR products were visualized via 2% agarose gel electrophoresis to confirm target bands and exclude non-specific amplification. Amplicons were then purified using the Monarch DNA Gel Extraction Kit (New England Biolabs, MA, USA). Purified products were used for library construction and sequenced on the Illumina NovaSeq platform (paired-end) (Illumina, San Diego, CA, USA).

Paired-end sequencing reads were generated by high-throughput sequencing. Based on the overlap between paired-end (PE) reads, Trimmomatic (https://trimmomatic.com/, accessed on 1 August 2025) and FLASH (Version 1.2.1.1) software were applied for data optimization. The optimization procedure included quality control, read merging, filtering, dereplication, and sequence orientation correction. The optimized sequences were further processed using Usearch (version 11.0, http://drive5.com/uparse/, accessed on 1 August 2025). Operational Taxonomic Unit (OTU) clustering was performed on the sequences at a 97% sequence similarity threshold. Sequence alignment and taxonomic annotation were carried out against the Silva database (Release 138, http://www.arb-silva.de, accessed on 1 August 2025) at different taxonomic ranks. Sequencing and data annotation were provided by Chongqing Bonoheng Biotechnology Co., Ltd. (Chongqing, China).

### 2.4. Data Statistics and Analysis

Bacterial Alpha diversity indices, including Sobs, Shannon, Simpson, Ace, Chao, and Coverage, were calculated using the vegan package in R (v4.4.1). Group differences were evaluated using the Kruskal-Wallis rank-sum test. To assess differences in community structure, Principal Coordinate Analysis (PCoA) based on Bray-Curtis distances was conducted. Additionally, Permutational Multivariate Analysis of Variance (PERMANOVA) was performed to determine statistical significance. All visualizations were generated using the ggplot2 package in R (v4.4.1). The Kruskal-Wallis rank sum test, coupled with false discovery rate (FDR) correction for multiple comparisons, was performed to analyze species with statistically significant intergroup differences at the phylum and genus taxonomic levels. The significance threshold was set at *p* = 0.05.

Interactions within bacterial communities were investigated using co-occurrence network analysis. Networks were constructed using the SparCC algorithm, which infers significant co-existing relationships while eliminating the biases inherent in compositional data. Before analysis, the OTU table was filtered to retain only OTUs present in at least 10% of samples with an average relative abundance of 0.01%. SparCC correlation analysis was performed with a pseudo-count of 1 and 20 iterations. Two-sided significance was estimated via 100 bootstraps, and *p*-values were corrected using the Benjamini–Hochberg method. Network edges were initially filtered based on the following criteria: |SparCC *r*| ≥ 0.20, FDR-adjusted *p* < 0.10, and a co-occurrence proportion ≥0.05. If these strict criteria yielded insufficient edges, thresholds were adaptively relaxed (minimum limits: |*r*| ≥ 0.05, FDR < 0.30, and co-occurrence proportion ≥0.02) to ensure retention of at least the 300 strongest correlations. The final network comprised the top 2000 edges ranked by |*r*|. Isolated nodes were removed, and the network was visualized using Gephi (Version 0.10.1). Community assembly mechanisms were analyzed by fitting the Neutral Community Model (NCM) using the stats4 package in R (v4.4.1). Additionally, niche breadth was calculated using the spaa package. Microbial functional prediction was performed using the FAPROTAX (Version 1.2.11) software to analyze biochemical cycle functions in different samples.

## 3. Results

### 3.1. Analysis of Microbial Sequencing Results

**Figure 2:**
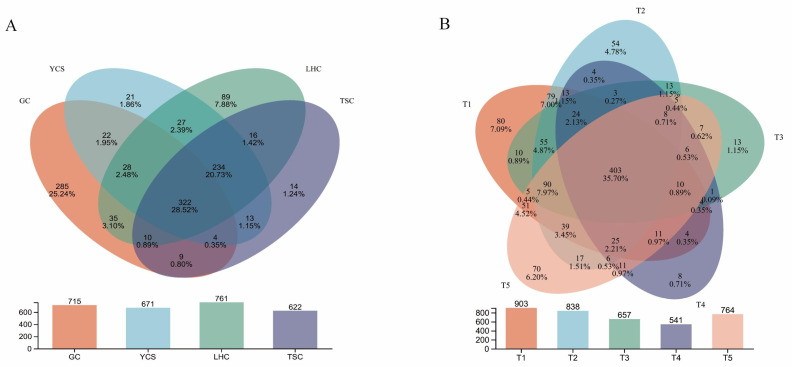
Microbial OTU changes at spatial (**A**) and temporal (**B**) scales.

A total of 3,337,242 effective sequences were obtained from 60 samples (*n* = 60). Venn diagrams were constructed to evaluate the similarity and overlap of Operational Taxonomic Units (OTUs) among groups (Figure 2). Spatially, a total of 1129 OTUs were identified (Figure 2A). The biofilter (LHC) exhibited the highest OTU richness (761 OTUs), while the gut (GC) contained the highest number of unique OTUs (285). A core set of 322 OTUs was shared across all four zones. Notably, GC and LHC shared 35 unique OTUs, exceeding the number of shared species between any other two zones. Temporally, a total of 1129 OTUs were identified (Figure 2B). The 403 OTUs were shared across all five time points, indicating that the core microbiome remained relatively stable within the system. Additionally, total OTU richness exhibited a trend of initially decreasing and subsequently increasing over the study period.

### 3.2. Species Diversity Analysis

**Figure 3:**
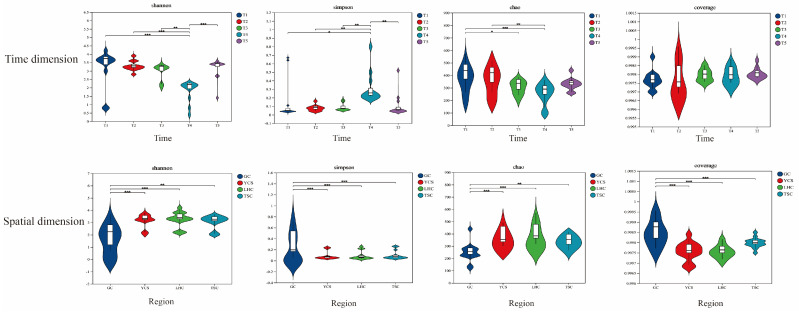
Alpha diversity indices of microbial community. Note: * indicates *p* < 0.05, ** indicates *p* < 0.01, *** indicates *p* < 0.001.

Alpha diversity indices (Shannon, Simpson, Chao, and coverage) revealed significant spatiotemporal differences (Figure 3). Temporally, the Shannon and Chao indices exhibited similar patterns: an initial decrease leading to minimum values at T4, followed by a significant increase at T5. Conversely, the Simpson index followed an inverse trend, peaking at T4 and declining significantly at T5. The coverage index remained stable throughout the study period (T1–T5), with no significant fluctuations. Spatially, distinct patterns emerged between the gut and the environmental samples. Diversity indices in the GC zone differed significantly from those in the YCS, LHC, and TSC zones. However, no significant differences were observed among the three environmental zones (YCS, LHC, and TSC) themselves.

**Figure 4:**
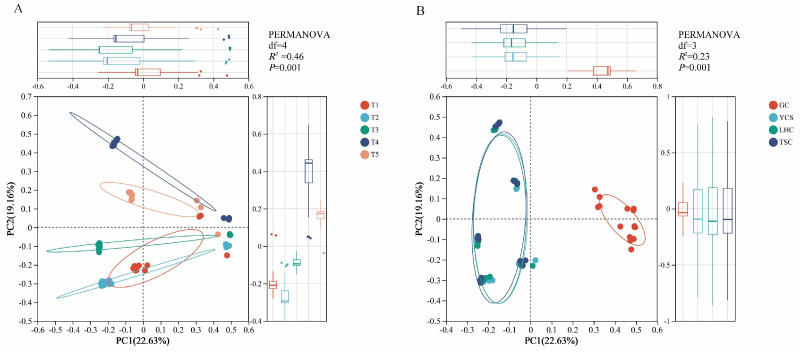
Principal coordinate analysis of microbial community at spatial (**A**) and temporal (**B**). Note: The ellipses represent the 95% confidence interval around the center point; df: degrees of freedom.

Principal Coordinate Analysis (PCoA) based on Bray-Curtis distances, verified by PERMANOVA, revealed significant spatiotemporal variations in microbial community structure (Figure 4). The first two axes accounted for a cumulative 41.79% of the variation (PCoA1: 22.63%, PCoA2: 19.16%). Temporally, sample points from different months showed distinct separation and significant differences (*R*^2^ = 0.46, *p* = 0.001) (Figure 4A). Spatially, the YCS, LHC, and TSC zones exhibited high overlap, reflecting a high degree of similarity among environmental sites. In contrast, the GC zone samples were distinctly separated from these environmental zones (*R*^2^ = 0.23, *p* = 0.001) (Figure 4B). These results confirm that both sampling time and location significantly influence microbial community assembly within the system.

### 3.3. Microbial Community Structure and Composition

**Figure 5:**
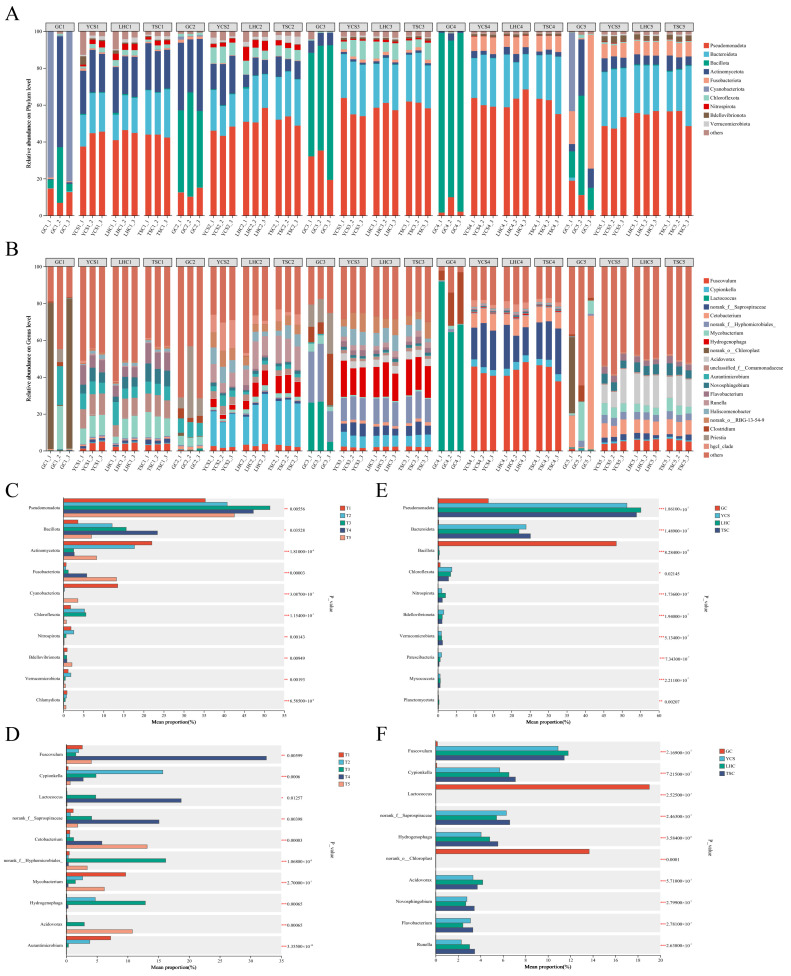
Relative abundance and significant difference analysis of microbial community. Taxonomic composition displaying the top 10 phyla (**A**) and the top 20 genera (**B**). The differences in the composition of the microbial communities between the phyla (**C**) and the genera (**D**) on the time scale. The differences in microbial communities between the phyla (**E**) and the genera (**F**) at the spatial scale. Note: * indicates *p* < 0.05, ** indicates *p* < 0.01, *** indicates *p* < 0.001.

Taxonomic composition was visualized using stacked bar charts displaying the top 10 phyla and top 20 genera (Figure 5A,B). The species composition of bacterial communities remained relatively stable overall at the phylum and genus levels. Relative abundances exhibited significant spatiotemporal fluctuations. A distinct compositional divergence was observed between the GC zone and the environmental zones (YCS, LHC, and TSC). Spatially, Pseudomonadota, Bacteroidota, and Actinomycetota were the dominant phyla in the environmental samples. In contrast, Bacillota was the predominant phylum in the GC zone. Temporally, trends varied by zones. In the environmental zones, Actinomycetota abundance gradually decreased from T1 to T4 before increasing at T5. Pseudomonadota and Bacteroidota remained stable, whereas Fusobacteriota showed an overall increasing trend. Conversely, within the GC zone, Bacillota abundance increased from T1 to T4 but declined at T5.

At the genus level, *Fuscovulum*, *Cypionkella*, *norank_f__Saprospiraceae*, *Hydrogenophaga*, and *Acidovorax* were dominant in the environmental zones (YCS, LHC, and TSC). Conversely, the GC zone was dominated by *Lactococcus*, *Clostridium*, and *Priestia*. Certain genera, including *Cetobacterium*, *norank_f__Hyphomicrobiales*, *Mycobacterium*, and *Aurantimicrobium*, were prevalent across all four zones. Temporally, distinct fluctuation patterns were observed. In the environmental zones, *Fuscovulum* and *norank_f__Saprospiraceae* increased significantly at T4. *Cypionkella* rose from T1 to T2 before declining, while *Hydrogenophaga* peaked at T3. A significant spike in *Acidovorax* occurred at T5. In the GC zone, *Lactococcus* and *Clostridium* abundance increased during T3–T4, peaking at T4, whereas *Priestia* peaked earlier at T3. Among the ubiquitously distributed genera, *Cetobacterium* increased significantly at T4 and T5, with the most pronounced increase occurring in the GC zone at T5. *norank_f__Hyphomicrobiales* increased significantly at T3. *Mycobacterium* gradually decreased during the T1–T4 period but significantly increased again at T5. Finally, *Aurantimicrobium* exhibited a continuous decline throughout the study period.

A Kruskal-Wallis rank-sum test identified the major differential taxa driving spatiotemporal variations in community structure (Figure 5C–F). Temporally, significant differences were observed in the phyla Pseudomonadota, Bacillota, Actinomycetota, and Fusobacteriota. At the genus level, specific taxa were significantly increased at distinct time points: *Mycobacterium* and *Aurantimicrobium* at T1; *Cypionkella* at T2; *norank_f__Hyphomicrobiales* and *Hydrogenophaga* at T3; *Fuscovulum*, *Lactococcus*, and *norank_f__Saprospiraceae* at T4; and *Cetobacterium* and *Acidovorax* at T5. Spatially, differential phyla included Pseudomonadota, Bacteroidota, Bacillota, and Chloroflexota. At the genus level, *Lactococcus* and *norank_o__Chloroplast* were significantly increased in the GC zone. Conversely, the environmental zones (YCS, LHC, and TSC) were characterized by significantly increased abundances of *Fuscovulum*, *Cypionkella*, *norank_f__Saprospiraceae*, *Hydrogenophaga*, *Acidovorax*, *Novosphingobium*, *Flavobacterium*, and *Runella*.

### 3.4. Microbial Community Assembly Mechanisms

**Figure 6:**
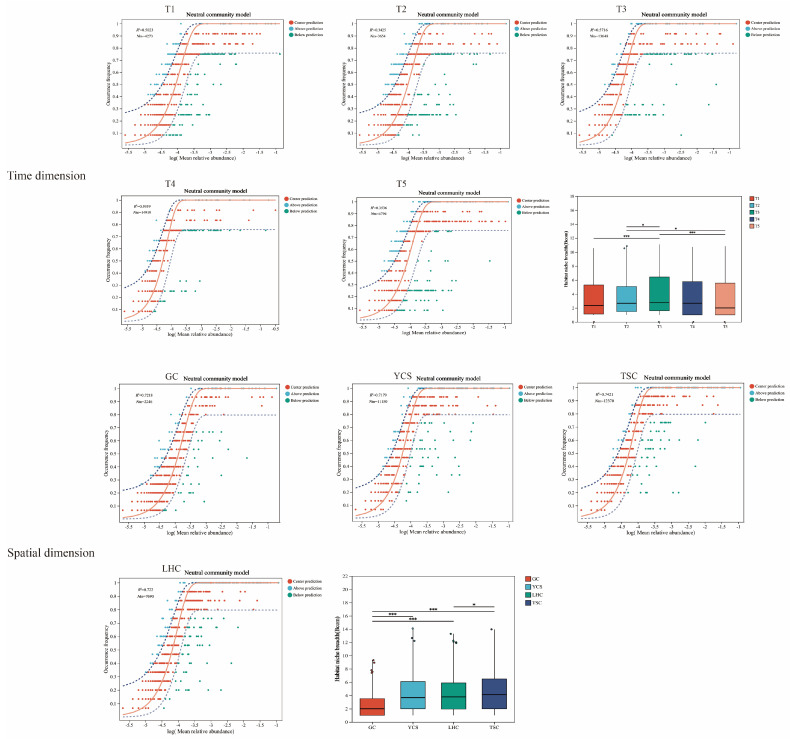
Comparison of neutral community model analysis and niche width of microbial community. Note: The solid line represents the best fit line of the model, the dashed lines indicate the upper and lower bounds of the 95% confidence interval of the model predictions, and Nm represents the product of the average total number of individuals of species (N) in the samples and the migration rate (m). Note: * indicates *p* < 0.05, *** indicates *p* < 0.001.

To elucidate the assembly mechanisms and niche dynamics of bacterial communities, we investigated the random and deterministic drivers in community formation and the ecological niche characteristics (Figure 6). The Neutral Community Model (NCM) was applied to quantify the influence of stochastic processes. Additionally, niche breadth comparisons were utilized to assess the functional adaptability and responsiveness of microbial populations to environmental fluctuations across different zones.

The *R*^2^ value of the NCM quantifies the goodness of fit. Higher values indicate a stronger influence of stochastic processes on community assembly. The parameter Nm represents the number of individuals migrating between locations per generation, with a higher Nm value indicating a higher migration rate. Increased migration of individuals between locations results in a more homogenous distribution of species across these sites.

Temporally, NCM fitting results (T1–T5) yielded *R*^2^ values of 50.23%, 34.25%, 57.16%, 59.59%, and 35.36%, respectively. Corresponding Nm values were 4273, 3654, 13,648, 14,919, and 4794. The elevated *R*^2^ and Nm values observed during T3–T4 indicate that species distribution was more homogenous and primarily governed by stochastic processes during this period. Spatially, *R*^2^ values across the four zones were consistently high (72.18%, 71.79%, 74.21%, and 72.20%), suggesting that stochastic processes played a significant role in shaping community structure throughout the system. However, migration patterns differed. The calculated Nm values were 2246, 11,150, 12,370, and 7690, respectively. Lower Nm values in the GC and LHC zones indicate restricted species dispersal in these compartments. Conversely, higher Nm values in the YCS and TSC zones imply weaker dispersal limitation and higher migration rates.

Furthermore, niche breadth refers to the sum of various resources utilized by a species (or other biological unit) within a community. The broader a species’ ecological niche, the less specialized it is, tending toward being a generalist species. The narrower a species’ ecological niche, the more it tends to be a specialist species. Temporally, niche breadth peaked at T3, suggesting that the microbial community was dominated by generalists during this period. Spatially, the GC and LHC zones exhibited narrower niche breadths, reflecting a tendency towards specialization. In contrast, the YCS and TSC zones displayed wider niches, indicating a prevalence of generalists.

### 3.5. Co-Occurrence Network Analysis

Co-occurrence networks were constructed to elucidate the complexity of microbial interactions across spatiotemporal scales. Nodes were distinctively colored to represent the top 15 phyla and five major modules. Topological analysis revealed significant variations in network structure across both time and space.

**Figure 7:**
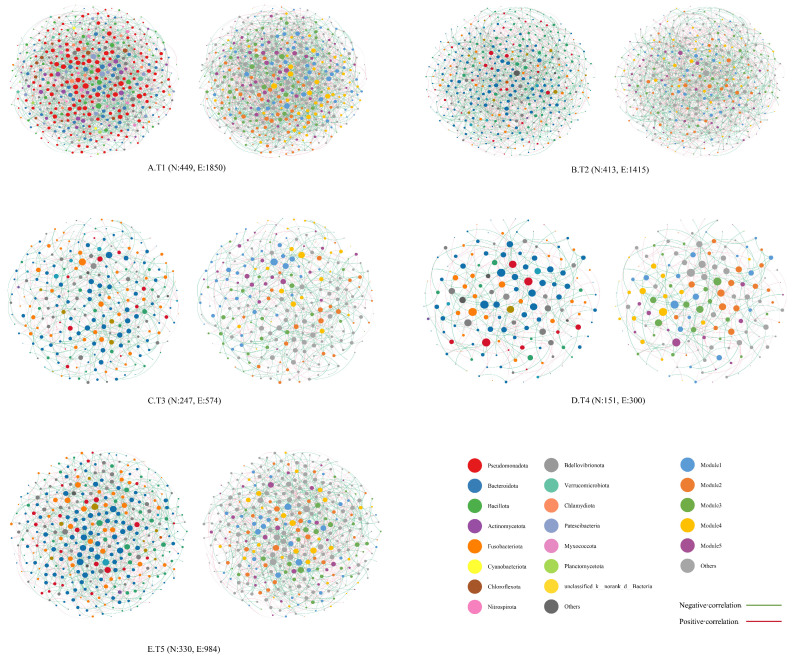
Co-occurrence network analysis of microbial community at temporal scales. Note: In (**A**–**E**), the left panel shows node colouring based on phylum level, while the right panel shows node colouring based on modularity class; larger node sizes indicate larger average degree; N is nodes; E is edges.

Temporally, network composition shifted from Pseudomonadota dominance at T1 to Bacteroidota prevalence during T2–T5 (Figure 7). In terms of topology (Table 1), T1 exhibited the highest complexity (1850 edges; 449 nodes) and interaction intensity (average degree: 8.241) but the lowest modularity. In contrast, T4 displayed the most structured architecture, recording peak values for network diameter (8), modularity (0.477), graph density (0.026), average clustering coefficient (0.035), and average path length (3.785). Furthermore, negative correlations were predominant across all periods (T1–T5), with proportions ranging from 0.5279 to 0.583.

**Figure 8:**
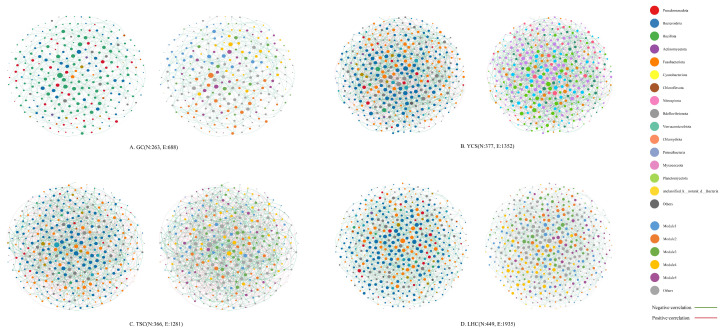
Co-occurrence network analysis of microbial community at spatial scales. Note: In (**A**–**D**), the left panel shows node colouring based on phylum level, while the right panel shows node colouring based on modularity class; larger node sizes indicate larger average degree; N is nodes; E is edges.

Spatially, network nodes in the GC zone were dominated by Bacillota (Figure 8). In contrast, the environmental zones (YCS, LHC, and TSC) were dominated by Bacteroidota and Fusobacteriota. Regarding topology (Table 2), the LHC zone exhibited the highest complexity (11,935 edges; 449 nodes) and interaction intensity (average degree: 8.619), despite having the lowest modularity. Conversely, the GC zone displayed the highest modularity (0.402) and average path length (3.542). The TSC zone recorded the highest average clustering coefficient (0.023). Network diameter and graph density showed no significant spatial variation. Consistent with temporal trends, negative correlations predominated across all four locations, with proportions ranging from 0.5628 to 0.5732.

### 3.6. FAPROTAX Functional Prediction

**Figure 9:**
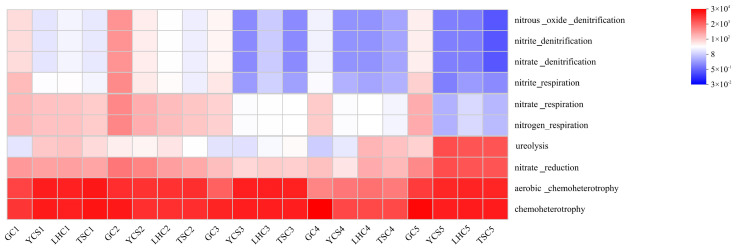
FAPROTAX Functional Prediction. Note: The *x*-axis represents sample names and the *y*-axis represents function names. The color gradient of the blocks illustrates the variation in function abundance across samples, with the numerical values corresponding to the color gradient shown on the right side of the graph.

To investigate the variations in microbial functional genes within the industrial recirculating aquaculture system throughout the experimental period, FAPROTAX functional gene prediction was performed (Figure 9). As illustrated in the figure, the community was dominated by chemoheterotrophy and aerobic chemoheterotrophy. These two genes represent the ability to break down organic compounds for energy and the requirement for oxygen to break down organic compounds, respectively. These metabolic functions constitute the fundamental basis for microbial survival. In the environmental zones (YCS, LHC, and TSC), genes associated with denitrification (nitrate, nitrite, and nitrous oxide) and respiration (nitrite, nitrate, and nitrogen) followed similar trends, characterized by a decline over time. In contrast, functions related to ureolysis and nitrate reduction increased significantly during the T5 period.

## 4. Discussion

### 4.1. Spatiotemporal Distribution Characteristics of Microbial Communities

Alpha diversity indices reflect microbial community stability and environmental adaptability [29]. In this study, significant temporal variations were observed. Specifically, the Shannon index reached a minimum at stage T4, while the Simpson index peaked. This divergence likely resulted from rapid fish growth and peak feed input during this period. The substantial accumulation of residual feed and feces increased nutrient levels such as nitrogen and phosphorus, while the enrichment of metabolic wastes created selective environmental pressure [30]. This pressure resulted in the mass elimination of microorganisms with low tolerance to high nutrient and pollutant levels, thereby significantly reducing species richness. Simultaneously, a small subset of microorganisms possessing efficient organic degradation capabilities proliferated rapidly and established dominance, leading to a marked increase in community dominance indices [31]. Subsequently, the rise in the Shannon index and the decline in the Simpson index at stage T5 suggest system resilience. The degraders enriched during T4 likely removed organic pollutants through metabolic processes, creating conditions favorable for the recovery of community richness [32]. Spatially, distinct patterns emerged between the gut and environmental zones. The GC zone microbiome is directly shaped by host physiology and diet, creating a unique, stable niche. In contrast, the YCS, LHC, and TSC zones are interconnected by water circulation. This high connectivity promotes uniform nutrient distribution, resulting in similar diversity profiles across these environmental compartments.

Regarding community composition, the environmental zones (YCS, LHC, and TSC) were dominated by Pseudomonadota, Bacteroidota, and Actinomycetota. Consistent with previous findings, these adaptable taxa play crucial roles in organic matter degradation and nitrogen transformation [33]. Conversely, the GC zone was dominated by Bacillota, a phylum linked to host nutrient metabolism [34]. These results highlight the adaptive responses of microbial communities to distinct habitats. Furthermore, the T4 stage emerged as a critical period. In the environmental zones, the relative abundances of *Fuscovulum* and *norank_f__Saprospiraceae* increased significantly. This shift likely occurred in response to elevated nutrient loads, as these taxa facilitate the degradation of nitrogenous substances [35,36]. Concurrently, the surge of *Lactococcus* in the GC zone suggests this period coincided with rapid fish growth, where this genus enhances nutrient absorption [37]. Therefore, aquaculture managers should prioritize water quality regulation during this critical phase.

### 4.2. Mechanisms of Microbiome Assembly

In the study of community assembly mechanisms, neutral theory and niche theory are the two most widely applied frameworks. Neutral theory posits that community formation is driven by stochastic processes, such as species birth, death, migration, and dispersal limitation [38]. Conversely, niche theory suggests that community formation is influenced by deterministic factors such as environmental conditions, habitat heterogeneity, and interspecies interactions. These factors determine the presence and abundance of species [39]. Existing studies indicate that deterministic and stochastic processes act conjointly on microbial community formation [40]. However, their relative importance depends on environmental types, conditions, and biological characteristics [41].

In this study, stochastic processes dominated during the T3 and T4 stages, as evidenced by significantly elevated *R*^2^ and Nm values. This dominance is likely attributable to rapid fish growth, which induced greater hydrodynamic disturbance, thereby accelerating random microbial dispersal. Furthermore, the high-nutrient environment facilitated species migration and dispersal, thereby resulting in higher species abundance and community consistency. Spatially, NCM goodness of fit exceeded 70% across all zones (YCS, LHC, TSC, and GC), further suggesting that stochastic processes played a leading role in community assembly. However, the Nm values in the YCS and TSC zones were significantly higher than those in the GC and LHC zones. This is likely because the YCS and TSC serve as zones with active water exchange, offering lower resistance to microbial dispersal via water flow and thus facilitating smoother species migration. In contrast, the GC zone possesses physical barriers that restrict the stochastic influx of external microorganisms [42]. Similarly, in the LHC zone, spatial obstruction by carrier media reduces the efficiency of random species diffusion [43]. This ultimately resulted in restricted microbial diffusion in these two zones.

Regarding niche breadth, the value observed during the T3 stage was significantly higher than in other stages, which aligns with the finding that stochastic processes dominated the NCM results for this period. Moreover, generalist species possess broad environmental adaptability and are more capable of occupying niches through random dispersal [44]. This also confirms the ecological niche characteristics of microbial communities during periods of strong random processes. Spatially, the niche breadth values in the GC and LHC zones were significantly lower than those in the YCS and TSC zones. This may be because the GC and LHC zones act as the fish gut and biofilter, respectively, creating unique attachment habitats. In contrast, the YCS and TSC habitats exhibit high homogeneity and connectivity, allowing generalist species to survive more easily under conditions dominated by random dispersal.

### 4.3. Co-Occurrence Network Characteristics of Microbial Communities

Co-occurrence networks provide intuitive insights into microbial interactions, where subtle topological shifts often mirror community responses to dynamic environmental conditions [45]. In this study, the microbial community network structure exhibited distinct phasic characteristics across the temporal dimension. The T1 network was characterized by high complexity and interaction intensity (average degree = 8.241), yet exhibited the lowest modularity. Taxonomically, this stage was dominated by Pseudomonadota. These patterns likely resulted from significant environmental fluctuations typical of early aquaculture stages, which drove rapid succession and the establishment of broad, albeit loose, associations [46]. The prevalence of Pseudomonadota reflects its broad adaptability and strong competitiveness in such conditions [47]. By stage T4, the network topology shifted to exhibit the highest values for modularity (0.477), average path length, and clustering coefficient, concurrent with a transition to Bacteroidota dominance. This suggests that as the system stabilized, the microbial community gradually differentiated into several modules that are highly relevant in function or ecological niche [48]. These modules were defined by enhanced internal cooperation and reduced inter-module connectivity. Bacteroidota likely formed more stable and specific functional clusters by participating in core functions such as organic matter degradation [49].

Spatially, microbial network structures varied significantly across compartments, driven by distinct niche adaptations and functional demands. The GC zone network was dominated by Bacillota. It exhibited the highest modularity (0.402) and a relatively long average path length. These metrics indicate a highly organized and functionally differentiated community, tailored to host-specific processes such as digestion and immune regulation [50]. Conversely, the LHC zone displayed the highest network complexity (11,935 edges) yet the lowest modularity. This structure likely results from the biofilter’s role as the primary water treatment unit. Tasked with multiple functions like ammonia nitrogen transformation and organic matter decomposition [51], the biofilter recruits diverse taxa with high diversity and broad metabolic capabilities, creating a dense, intersecting interaction network [52]. In comparison, the YCS and TSC zones shared similar network architectures, both dominated by Bacteroidota and Fusobacteriota. Notably, across both temporal and spatial scales, negative correlations consistently outnumbered positive ones within the microbial communities. This prevalence of negative interactions suggests that the mandarin fish recirculating aquaculture system acts as a competition-dominated ecosystem [53]. Within this system, distinct habitats partition microorganisms into separate niches, thereby intensifying negative interactions.

### 4.4. Faprotax Function Prediction and Nitrogen Conversion

Nitrogen plays a very important role in RAS. It can significantly affect fish growth and water quality [54]. In this study, genes associated with chemoheterotrophy and aerobic chemoheterotrophy exhibited the highest abundance. This pattern aligns with the ecological attributes of RAS, a system typically characterized by high organic loads and high C/N ratios [55]. Inputs such as feed, fish excretion, and the decomposition of uneaten particles generate substantial organic matter, establishing a metabolic foundation primarily driven by heterotrophy. This finding corroborates the co-occurrence network analysis, which identified a competition-dominated community. Specifically, it suggests that competition for organic carbon sources acts as a key ecological force shaping community structure [56]. During the T4 period, the relative abundances of *Fuscovulum* and *norank_f__Saprospiraceae* in the water column increased significantly. This indicated that nitrification was enhanced at this stage, which facilitated the formation of nitrate [35,36]. In contrast, during the T5 period, the relative abundance of *Acidovorax* increased significantly. Existing studies have demonstrated that *Acidovorax* is a denitrifying bacterium [57], which can sequentially reduce nitrate to N_2_. This suggested that denitrification might dominate the nitrogen transformation process in the system at this stage to cope with the nitrate produced during the T4 period. However, the functional capacities of urea hydrolysis and nitrate reduction were significantly enhanced during the T5 period. This may be attributed to the accumulation of urea excreted by cultured fish in the late stage, which drove the microbial community to shift to a nitrogen transformation pathway dominated by urea hydrolysis and nitrate reduction. Nevertheless, this metabolic shift risks the concomitant accumulation of nitrite and ionic ammonia. Therefore, attention should also be paid to the regulation of aquaculture water quality during the T5 period.

### 4.5. Recommendations and Future Research Directions

This study systematically elucidated the spatiotemporal dynamic characteristics of microbial community structure in RAS for mandarin fish, which holds certain guiding value for practical production. Managers should pay close attention to the T4 stage and strengthen water quality monitoring and environmental regulation. This is to avoid water quality deterioration and disease outbreaks caused by the homeostasis imbalance of microbial communities. Meanwhile, it is recommended that the managers supplement relevant functional bacteria, such as nitrifying bacteria and *Bacillus* spp., in a targeted manner at the T5 stage to enhance the nitrogen removal capacity of the system. Future research can carry out the isolation, screening and in situ application efficacy verification of core nitrogen-cycling functional bacteria and elucidate the microbial interaction mechanisms among different functional units. In addition, it is also essential to explore the correlation patterns between microbial community dynamics throughout the whole culture cycle and growth performance as well as disease occurrence of mandarin fish. This helps to establish a technical system for health early warning and precise regulation of RAS based on microbial community characteristics.

## 5. Conclusions

(1) The microbial community structure within the mandarin fish RAS exhibited significant variations across both temporal and spatial dimensions. During the T4 stage, the relative abundances of *Fuscovulum* and *norank_f__Saprospiraceae* in the YCS, LHC, and TSC zones increased significantly to facilitate the degradation of nitrogenous substances. Concurrently, the significant increase in the relative abundance of *Lactococcus* in the GC zone indicated rapid growth of the mandarin fish, necessitating this taxon to enhance nutrient absorption. However, strict water quality regulation is required during this stage to prevent excessive nitrogen loading within the system.

(2) The assembly of microbial communities in the mandarin fish RAS was primarily governed by stochastic processes. Variations in niche breadth across temporal and spatial scales suggest that hydraulic connectivity and environmental selection conjointly shaped the microbial community structure. Furthermore, microbial interactions were predominantly characterized by competition. The network structure evolved from a loose configuration toward a modular architecture over time. Spatially, the networks exhibited differentiation driven by distinct functional requirements.

(3) The core metabolic function of microorganisms in the mandarin fish RAS was chemoheterotrophy. The nitrogen transformation pathway shifted from being dominated by denitrification to ureolysis and nitrate reduction as the culture progressed, indicating a need to monitor the risks of nitrite and ammonia nitrogen accumulation during the late culture stages.

## Figures and Tables

**Table 1:** Topological characteristics of microbial co-occurrence networks at temporal scales.

Groups	Nodes	Edges	Average Degree	Network Diameter	Density	Modularity Index	Average Clustering Coefficient	Average Network Distance	Positive Correlation	Negative Correlation
T1	449	1850	8.241	6	0.018	0.313	0.025	3.132	0.4335	0.5665
T2	413	1415	6.852	6	0.017	0.349	0.021	3.327	0.417	0.583
T3	247	574	4.648	8	0.019	0.45	0.034	3.773	0.4721	0.5279
T4	151	300	3.974	8	0.026	0.477	0.035	3.785	0.4333	0.5667
T5	330	984	5.964	7	0.018	0.371	0.02	3.435	0.4278	0.5722

**Table 2:** Topological characteristics of microbial co-occurrence networks at spatial scales.

Groups	Nodes	Edges	Average Degree	Network Diameter	Density	Modularity Index	Average Clustering Coefficient	Average Network Distance	Positive Correlation	Negative Correlation
GC	263	688	5.232	6	0.02	0.402	0.012	3.542	0.4302	0.5698
YCS	377	1352	7.172	6	0.019	0.336	0.02	3.225	0.4268	0.5732
TSC	366	1281	7	6	0.019	0.347	0.023	3.241	0.4372	0.5628
LHC	449	1935	8.619	5	0.019	0.292	0.02	3.065	0.4295	0.5705

## Data Availability

The original contributions presented in this study are included in the article. Further inquiries can be directed to the corresponding author.
